# Thioredoxin-Interacting Protein (TXNIP) in Gestational Diabetes Mellitus

**DOI:** 10.3390/metabo15060351

**Published:** 2025-05-26

**Authors:** Ioanna Kokkinopoulou, Anna Papadopoulou

**Affiliations:** Laboratory of Clinical Biochemistry, University General Hospital ‘Attikon’, Medical School, National and Kapodistrian University of Athens, 12462 Athens, Greece; iwanna-k@med.uoa.gr

**Keywords:** TXNIP, GDM pregnancy, oxidative stress, inflammation, placental dysfunction

## Abstract

**Background:** Thioredoxin-interacting protein (TXNIP) is a major inhibitor of the thioredoxin (TRX) antioxidant system and an important player in the development and aggravation of intracellular oxidative stress. Although first recognized as a metabolic regulator, recent studies have identified the multifaceted role of this protein in other molecular pathways involving inflammation, apoptosis, and glucose metabolism. **Methods**: This review aims to highlight the importance of TXNIP in diabetes-related pathophysiology and explore the existing evidence regarding TXNIP’s role in GDM-associated pathogenetic mechanisms, revealing common regulatory pathways. **Results:** Among other complex diseases, TXNIP has been found upregulated in diabetic pancreatic beta cells, thus contributing to diabetes pathogenesis and its related complications. In addition, depletion of TXNIP has been shown to decrease the negative consequences of excessive stress in various cellular systems and diseases, pointing towards a potential therapeutic target. In line with these findings, TXNIP has been investigated in the pathogenesis of Gestational Diabetes Mellitus (GDM), a common pregnancy complication affecting the mother and the neonate. Overexpression of TXNIP has been found in GDM placentas or trophoblast cell lines mimicking GDM conditions and has been associated with key dysregulated mechanisms of GDM pathophysiology, like oxidative stress, inflammation, apoptosis, impaired autophagy, altered trophoblast behavior, and placental morphology. Interestingly, TXNIP has been found upregulated in GDM maternal serum and downregulated in umbilical cord blood, indicating potential compensatory protective mechanisms to GDM-related oxidative stress. **Conclusions:** Due to its contribution to the regulation of critical cellular processes such as inflammation, metabolism, and apoptosis, TXNIP finds its place in the pathophysiology of gestational diabetes through a currently limited number of scientific reports.

## 1. Introduction

Thioredoxin-interacting protein (TXNIP) is recognized as a negative physiological regulator of the maintenance of the cellular reducing environment [1]. Oxidative stress enhances TXNIP expression in specific cell compartments, which subsequently triggers the stimulation of apoptotic and inflammatory responses through molecular changes, which will be elucidated. Under excessive stress conditions, TXNIP is overexpressed, giving rise to more intense cellular responses, resulting in the activation of caspases and/or pro-inflammatory molecule signaling [2]. Furthermore, TXNIP depletion by endogenous or exogenous intervention may smooth or decrease the negative consequences of excessive stress in diabetic beta cells and diabetic animal models [3]. This evidence argues for a potential new therapeutic target in diabetes. Gestational Diabetes Mellitus (GDM) is a common pregnancy complication associated with any degree of glucose intolerance first recognized during pregnancy [4]. Hyperglycemia travels in fetal circulation and may result in fetal overgrowth or adverse consequences for the mother and the neonate. Disturbed molecular pathways are associated with increased oxidative stress and inflammation [5]. The discovery that TΧNIP is a potential therapeutic target is a strong incentive for its further exploration in gestational diabetes, as proper patient management is crucial for the good health of the mother and the neonate. This review summarizes the published knowledge on the implications of TXNIP in apoptotic and inflammatory mechanisms in diabetes and discusses the results obtained from TXNIP studies in GDM.

## 2. TXNIP: Roles and Mechanisms of Action

TXNIP, alternatively known as thioredoxin-binding protein 2 (TBP-2), or vitamin D3 upregulated protein 1 [6], is a scaffolding protein that belongs to the α-arrestin family and interacts with multiple partners, such as thioredoxin (TRX) and mitogen-activated protein kinase (MAPK) [3]. In 2004, studies on HcB-19 mice, a mouse model of hyperlipidemia, showed that mutated TXNIP (Txnip-/-) resulted in increased fatty acid synthesis and cholesterol accumulation, attributing to TXNIP the role of a metabolic regulator [7]. Later functional and structural studies revealed the key roles of this molecule in a wide range of TXNIP-related molecular cascades implicated in cell oxidative stress and inflammation in the pancreas, liver, kidneys, skeletal muscle, and adipose tissues [1,8,9], occurring via TRX redox-dependent or redox-independent mechanisms [10,11,12].

### 2.1. TXNIP Structure

TXNIP consists of two arrestin domains at its amino terminus: N-arrestin (N-ARR) and C-arrestin; (C-ARR). Within N-ARR, TXNIP contains two SH3-binding domains (PxxP) that bind SH3 domains of kinases such as Src and ASK1, a Mitogen-Activated Protein Kinase Kinase Kinase (MAPKKK) [9]. Within C-ARR, there is an ITIM (immunoreceptor tyrosine-based inhibitory) sequence, around Tyr279, that recruits tyrosine phosphatases [9]. Nearby, a leucine-rich CRM1 motif centered on Leu294 mediates the binding of transcriptional factor hypoxia-inducible factor-1α (HIF-1α) and von Hippel–Lindau ubiquitin ligase (pVHL). Through this CRM1–HIF1α–pVHL complex, TXNIP promotes nuclear export and proteasomal degradation of HIF-1α [9]. The arrestin-like domains and the signaling motifs serve as a scaffold for protein–protein interactions, contributing to TXNIP’s redox-independent signaling functions.

**Figure 1:**
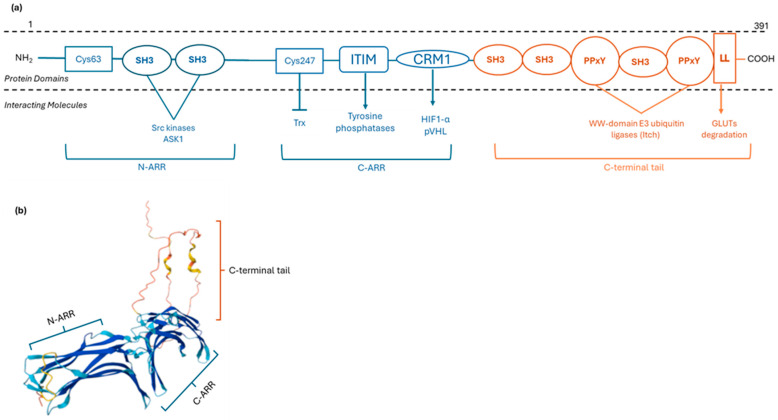
Schematic representation of the human TXNIP structure. (**a**) TXNIP contains two arrestin-like domains (N-ARR and C-ARR). In the N-ARR domain, SH3-binding PxxP motifs are present, facilitating protein–protein interactions. The C-ARR domain contains critical cysteine residues (Cys63, Cys247), as well as ITIM and CRM1 binding regions. The C-terminal tail includes two PPxY motifs, an LL motif, and additional SH3-binding PxxP motifs. (**b**) The 3D structure of TXNIP, as predicted by the AlphaFold database (UniProt ID: Q9H3M7). N-ARR: N-arrestin like domain; C-ARR: C-arrestin like domain; SH3: Src homology 3; ITIM: immunoreceptor tyrosine-based inhibitory; CRM1: chromosome maintenance region 1; LL: dileucine; GLUTs: Glucose Transporters; HIF1-α: hypoxia-inducible factor-1α; pVHL: von Hippel–Lindau ubiquitin ligase; Trx: Thioredoxin; ASK1; apoptosis signal-regulating kinase 1.

The C-terminal tail of TXNIP also harbors additional SH3 binding sequences that recruit other SH3-domain-containing proteins, thereby linking TXNIP into diverse signaling networks in the nucleus, the cytosol, and the plasma [9]. At TXNIP’s C-terminus tail, there are two conserved PPxY motifs and a dileucine (LL) endocytic motif. The PPxY motifs bind WW-domain E3 ubiquitin ligases, such as Itch, targeting TXNIP for ubiquitin-mediated proteasomal degradation [9]. The dileucine motif mediates clathrin-dependent endocytosis of membrane proteins bound by TXNIP, including class I glucose transporters (GLUT1,3,14,4) [13] (Figure 1). Although TXNIP is classified as a member of the α-arrestin protein family, its active cysteine residues that enable its interaction with TRX are not present in other family members, indicating that TXNIP has the distinct ability to modulate the cellular redox environment.

### 2.2. TXNIP/TRX Redox System

**Figure 2:**
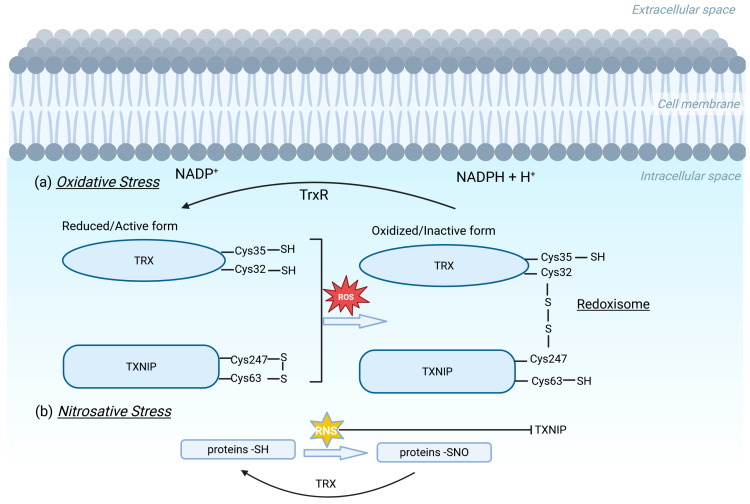
A schematic representation of TRX regulation by TXNIP under (**a**) oxidative and (**b**) nitrosative stress conditions. TRX alternates between its oxidized (inactivated) and reduced (activated) form, through the catalytic action of NADPH-dependent TrxR, thus regulating cellular redox homeostasis. Under excessive ROS production, a disulfide bond between TXNIP Cys247 and TRX Cys32 is formed, leading to the formation of redoxisome complex and inactivation of TRX. Under high RNS levels, TXNIP is downregulated, facilitating TRX-mediated denitrosylation, a critical process for maintaining cellular redox balance. ROS: reactive oxygen species; RNS: reactive nitrogen species; TrxR: thioredoxin reductase; Trx: Thioredoxin. Created in © 2025 BioRender. Kokkinopoulou, I. (2025) https://BioRender.com/5tmi5uq (accessed on 13 May 2025).

The TRX redox system consists of the TXNIP, the antioxidant protein TRX, the enzyme thioredoxin reductase (TRX-R), and the transporter of electrons nicotinamide adenine dinucleotide phosphate (NADPH) [14]. In mammalian cells, there are two isoforms of TRX: Thioredoxin 1 (TRX1)—which is localized in the cytosol, the plasma membrane, and the nucleus—and Thioredoxin 2 (TRX2), which is localized only in mitochondria [15]. TRX alternates between oxidized (inactivated) and the reduced (activated) states through catalysis by the NADPH-dependent TRX reductase [14]. In metabolically essential sites, rich in mitochondria, oxidized proteins or reactive oxygen species (ROS) are “captured” by the reduced form of TRX, thus protecting the cell from oxidative damage [16]. Interestingly, targeted disruption of the mouse thioredoxin gene is detrimental for differentiation and morphogenesis of the mouse embryo [17]. The oxidized form of TXNIP interacts with the reduced TRX using dithiol–disulfide exchange reactions between cysteine amino acids of both proteins, with the subsequent structural reorganization of TXNIP [18], formation of the TRX/TXNIP redox-complex, called “redoxisome”, and the inhibition of the reductive function of TRX [3,19] (Figure 2). Moreover, in vivo and in vitro studies focused on silencing the TXNIP gene or blocking it by antagonists or inhibitors, such as with small molecule drugs, peptides, and micro-RNAs, have confirmed the inhibitory effect of TXNIP on TRX redox activity, proposing TXNIP as a potential therapeutic target for various diseases [20,21,22,23,24,25,26]. On the other hand, excess nitric oxide (NO) suppresses TXNIP expression, thus allowing TRX to mediate denitrosylation, a critical process that protects cells from nitrosative stress [27].

### 2.3. TXNIP and Mechanisms of Action

Under normal conditions, low levels of cellular ROS are required for normal cellular signaling [28], where TXNIP acts as a rheostat to ensure an optimal balance of the cellular redox environment. Under chronic excessive intracellular ROS concentrations induced by high glucose, insulin, or other glycolytic metabolic intermediates [29,30,31,32,33]—TXNIP expression is upregulated and shuttles into mitochondria or cytosol, from the nucleus where it is normally located [34]. TXNIP overexpression has also been associated with activation of the unfolded protein response (UPR), also known as ER stress [9]. The effects of overexpressed TXNIP have been investigated in many different experimental animal models and tissues and it has been shown to contribute to human diseases like diabetes, ischemic diseases, intracerebral hemorrhage, neurodegenerative diseases, and cardiovascular diseases through redox-imbalance and inflammasome activation [8,14,20,35,36]. Starting from different departure molecules, like plasma membrane receptors or other intracellular scavengers, TXNIP overexpression stimulates various signaling pathways, giving rise to multiple responses such as the following: the induction of apoptotic related factors, the increased synthesis and secretion of inflammatory products like cytokines, and the generation of metabolic intermediates in glucose and lipid metabolism.

The action of TXNIP has been identified in different cellular compartments. In cytosol, TXNIP interacts with TRX1, and results in the dissociation of TRX1 from the apoptosis signal-regulating kinase 1 protein (ASK1), and consequently in the activation of p38 mitogen activated protein kinase pathway [36]. In mitochondria, TXNIP interacts with TRX2, the mitochondrial isoform of TRX, which results in the dissociation of TRX2 from the ASK1. This release generates mitochondrial ROS while ASK1 is phosphorylated and stimulates cyto C secretion from mitochondria into the cytosol [14,34,36]. Cyto C in turn activates Apaf-1 and promotes the apoptotic pathway through caspases, causing damage to various tissues, like the heart, kidneys, and lungs [36]. The effects of this signaling are counterbalanced by the anti-apoptotic AKT/Bcl-xL signaling, as shown in TXNIP depletion experiments [37].

Apart from the apoptotic process, the ASK1 activation pathway is shown to be responsible for the increased synthesis of inflammatory molecules like IL-1b and IL-18 in beta-cells or the vascular cell adhesion molecule 1 (VCAM-1) in endothelial cells, indicating a link between TXNIP oxidation and inflammation [38]. Indeed, recent works have revealed that high glucose and ROS concentrations as well as activated UPR may promote TXNIP-induced inflammation [12,39,40]. In that case, the nuclear factor-κB (NF-κB) translocates from the cytosol to the nucleus and promotes the transcription of the *NLRP3* gene, along with the synthesis of pro-inflammatory cytokines [41]. Furthermore, in mitochondria, TXNIP dissociates from TRX2 and interacts physically with NLRP3 in an ROS-sensitive manner in cells like macrophages [12,31,42,43,44]. The complex TXNIP-NLRP3 exits the mitochondria and forms the NLRP3 inflammasome, a multiprotein complex composed of NLRP3, ASC (apoptosis-associated speck-like protein), and pro-caspase-1 [37]. The inflammatory reaction proceeds with the release of IL-18 and IL-1b in the cytosol through caspase 1 activation [45]. Inflammasome activation is shown to be blocked in TXNIP-depleted models and inhibited by ROS inhibitors [3]. Moreover, caspase 1 promotes another means of programmed cell death, called pyroptosis, by cleavage of gadersmin D and the formation of membrane pores [45]. This phenomenon has been shown to be associated with diabetes, hypertension, and hyperlipidemia [46].

TXNIP also regulates various aspects of metabolism, especially glucose uptake, presumably through the regulation of glucose transporters (GLUTs) [47]. The elevation in glucose uptake enhances TXNIP expression in cells, which can further induce mRNA expression of Glut1 and excessive ROS production in mitochondria and cytosol, as shown in TXNIP-depleted HepG3 cells [36]. TXNIP modulates the expression and position of Glut1 to prevent glucose uptake by activating PTEN, which in turn inhibits AKT-PI3 signaling and downregulates glucose uptake [11]. On the other hand, glucose-stimulated TXNIP induction may be mediated by multiple pathways, as in the binding of the transcriptional complex MondoA:MAX and the carbohydrate response element-binding protein (ChREBP) to the carbohydrate response element located on the promoter of the TXNIP gene [48,49,50,51], as well as via a positive feedback loop involving the activation of ChREBP [48]. This activation is counterbalanced by the forkhead transcription factor O1 (FOXO1) and mammalian target of rapamycin (mTOR), which inhibit ChREBP transcriptional activity [52]. It has been shown that beta-cell-specific mTOR deficiency increases the expression levels of TXNIP and ChREBP, leading to severe reductions in b-cell survival [53]. Finally, the large increase in TXNIP by hyperglycemia may be limited by insulin, which acts as part of a negative feedback loop [54].

## 3. The Role of TXNIP in Diabetes

Chronic hyperglycemic conditions result in chronic excessive generation of ROS, disrupt the cellular antioxidant system, aggravate insulin sensitivity, and induce oxidative stress and inflammation [32,55]. Hyperglycemia and alterations in insulin secretion and sensitivity are, along with beta cell death, the hallmarks of diabetes [34,56,57]. Because of the plethora of insulin-mediated effects on target-organs and tissues, diabetes may enhance other secondary complications in organs and tissues like the kidneys, liver, skeletal muscle, retina, heart, and adipose tissues. The link between hyperglycemia and TXNIP in diabetes has been extensively documented in pancreatic beta cells and in insulin target tissues, involving mechanisms of glucose-stimulated insulin secretion and sensitivity, glucose uptake [48,58], beta cell apoptosis, and inflammation [59,60].

In a human islet microarray study in 2002, Shalev et al. [61] observed that TXNIP is the most significantly glucose-induced gene in diabetic b-cells, through a distinct carbohydrate response element (ChoRE) in the human TXNIP promoter [48,62]. Glucose-induced TXNIP expression in diabetes has also been observed in non-beta cells—like in primary human aortic endothelial cells in a T1D-like rat model [63] and in the skeletal muscle of prediabetic and diabetic patients [60]—confirming its contribution in glucose-induced secondary complications of diabetes. On the other hand, thioredoxin expression does not follow glucose-induced TXNIP changes but has an opposite effect to TXNIP on the progression of diabetes [8]. Interestingly, it has been reported that the overexpression of TXNIP inhibits the activity of the thioredoxin system, while overexpression of Trx1 in beta cells significantly decreases blood glucose levels in the diabetic context in a site- and cell-specific manner [8].

As described earlier, the effect of the increased expression of TXNIP in human and mice diabetic beta cells—either by high glucose levels or by glucose-induced ER stress—has been described via mitochondrial apoptotic and NLRP3 inflammasome signaling pathways. Thus, TXNIP overexpression through high glucose exposure has been associated with increased ROS accumulation [64], and activates beta cell apoptosis primarily by stimulating the dissociation of ASK1-Trx2 in mitochondria and the activation of the caspase 3 apoptotic pathway [34,54,62]. This apoptotic mechanism has also been identified in brain [65] and neurodegenerative diseases [66], in myocardial ischemia [67], and in diabetic retinopathy [68]. TXNIP-induced beta cell apoptosis has also been associated with pro-inflammatory cytokines. At the same time, TXNIP induction, either by glucose or by glucose-induced ER stress, may activate the NLRP3 inflammasome, caspase-1, and IL-1b synthesis in beta cells, resulting in their dysfunction [69,70]. In addition, the modulation of the angiogenic cytokine VEGF [54] and interaction with VCAM1 have been reported in human aortic endothelial cells in angiogenesis [68]. NLRP3 and TXNIP knockout mice have shown improved glucose tolerance and insulin sensitivity in a T2DM model [12]. The NLRP3 inflammasome directs the obesity-associated danger signal, giving rise to obesity-induced inflammation and insulin resistance [71].

As shown in diabetic mouse models, TXNIP deficiency activates antiapoptotic AKT/BCL-xL signaling [37], increases beta cell mass, and protects against diabetes [37,72]. Decreasing Txnip mRNA and protein levels in pancreatic MIN6 cells reduce reactive oxygen species levels and restore glucose-regulated insulin secretion [73]. TXNIP induces specific beta-cell microRNA expression, like miR-204 [74], and miR200 [75] which in turn block insulin production and epithelial–mesenchymal transition, respectively. TXNIP knockdown by small interfering RNA can overcome the diabetes-related pathologies of angiogenesis, cardiomyopathy, and renal injury, and alleviates the apoptosis and inflammation of retinal cells in diabetic mice [68], which indicates that TXNIP could act as a therapeutic target.

## 4. Gestational Diabetes Mellitus

Human pregnancy is a complicated process, involving changes in maternal environment as an adaptive response to the increasing nutrient requirements of the growing fetus [76]. As the pregnancy advances, insulin sensitivity gradually decreases [77], and this decrease is mediated by an increase in the levels of hormones and cytokines, among other factors [78]. In case maternal metabolism is not adapted to these needs, instead of a mild reduction, there is a vast decrease in insulin sensitivity, resulting in fetal hyperglycemia and overgrowth [79]. After delivery, when the umbilical cord is cut, maternal nutrient supply stops, and all newborns experience a transient drop in glucose levels. Newborns of diabetic mothers are less able to establish the mechanisms of serum glucose maintenance [80,81].

Gestational diabetes mellitus represents the most prevalent metabolic disorder among pregnant women and is assimilated to pre-T2DM. Prediabetes usually precedes overt diabetes and includes impaired fasting glucose and impaired glucose tolerance, and it is similar to diabetes in the context of disglycemia and hyperglycemic glucotoxicity [54]. GDM is associated with any degree of glucose intolerance first recognized during pregnancy and diagnosed between 24 and 28 weeks of gestation [82]. The diagnosis is made after a 2 h 75 g oral glucose tolerance test (OGTT), performed after overnight fasting on women not previously found to have overt diabetes or GDM during earlier testing in the pregnancy between 24 and 28 weeks of gestation [83,84]. Risk factors for the development of GDM include overweight/obesity, physical inactivity, advanced maternal age, family history of type 2 diabetes mellitus, ethnicity, genetic polymorphisms, and polycystic ovarian syndrome. GDM is linked to various short- and long-term health consequences, like neonatal macrosomia and type 2 diabetes, for both the mother and the child [82]. To prevent the fatal consequences of GDM, all GDM pregnancies are treated either by diet or insulin. Although the molecular processes underlying GDM pathophysiology remain unclear, there is strong evidence supporting the claim that GDM is characterized by increased oxidative stress and inflammation [84,85,86]. Maternal hyperglycemia-induced oxidative stress leads to higher release of oxidative stress markers in the circulation of women with GDM, which in turn can cause beta cell dysfunction and apoptosis, impaired insulin secretion, and insulin resistance. Oxidative stress leads to the activation of inflammatory cells and release of inflammatory mediators. Indeed, several studies have revealed that women with GDM exhibit elevated circulating levels of pro-inflammatory cytokines, including IL-1β, IL-6, and TNF-a, which not only further mediate the inflammatory response but also enhance the generation of ROS. This creates a feedback loop where oxidative stress and inflammation perpetually reinforce each other, exacerbating cellular damage and metabolic dysfunction.

The human placenta is a complex organ which separates fetal and maternal circulation. It consists of different types of cells, such as trophoblasts, syncytiotrophoblast, endothelial cells, stomal cells, and tissue-resident macrophages (Hofbauer cells) [86]. The syncytiotrophoblast is the transporting epithelium, exposed to maternal and fetal circulation [87,88]. During gestation, the placenta senses the maternal and fetal changes and modifies the expression and the activity of channels and receptors as well as the secretion of hormones, cytokines, or other signaling molecules. Some signals will return back to the mother as a feedback loop, while others like fetal insulin and glucose directly or indirectly affect the endothelium or tissue-resident macrophages (Hofbauer cells) in placenta [86]. The complexity of the human placental tissue and the impact of multiple biological and environmental factors influencing maternal glucose metabolism constitute a complex network of pathways critical for fetal growth. This back transport is increased in diabetic rats [89]. Furthermore, insulin receptors at different locations of the placenta preferentially activate different intracellular signaling pathways [86]. For instance, at the beginning of pregnancy, insulin may have a mitogenic effect on the trophoblast, whereas fetal insulin will stimulate metabolic processes within the endothelium.

Structural and morphological alterations have been observed in placentas delivered from GDM pregnancies, finally impairing placental efficiency [90,91]. Placenta derived from GDM women has increased weight along with enlarged surface areas of exchange on the syncytiotrophoblast and fetal endothelium side [84]. GDM placenta villi exhibit differentially expressed proteins associated with the development of insulin resistance, hypocalcemia, oxidative stress, placental transport capacity, and inflammation, as well as with other factors affecting normal fetal growth [85,86,92,93]. In diet-treated GDM, the amount of trophoblast insulin receptors is lower than in nondiabetic pregnancies, whereas in insulin-treated GDM, the placenta contains more insulin receptors [94]. Whether endothelial insulin receptors are also altered is unknown.

## 5. The Role of TXNIP in GDM Pathophysiology

**Figure 3:**
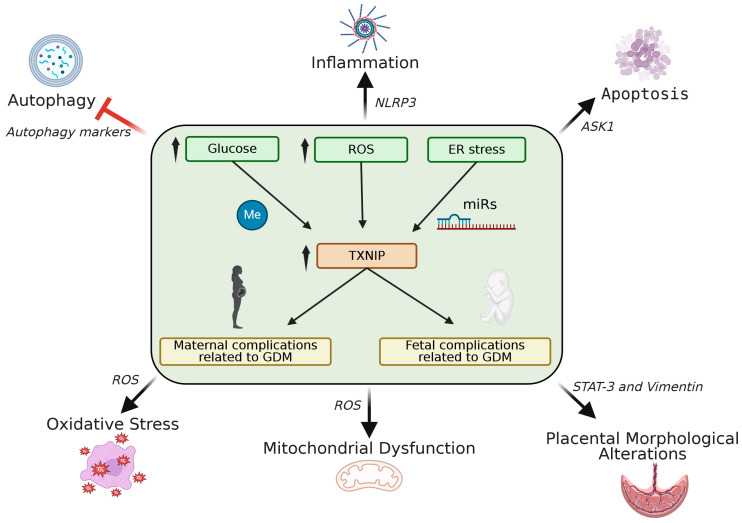
The role of TXNIP dysregulation in GDM-associated pathophysiological mechanisms. GDM-associated hyperglycemia, ROS generation, and ER stress induce TXNIP upregulation through modulation of TXNIP methylation status and/or upregulation of miRNAs targeting TXNIP. Hyperglycemia-induced TXNIP upregulation promotes oxidative stress and mitochondrial dysfunction through increased ROS generation, enhances apoptosis through ASK1 activation, induces inflammation through NLRP3 inflammasome activation, inhibits autophagy, and impairs trophoblast morphology and behavior through dysregulation of STAT-3 and Vimentin-related mechanisms, ultimately contributing to GDM-related maternal and fetal complications. Created in © 2025 BioRender. Kokkinopoulou, I. (2025) https://BioRender.com/cettpq6 (accessed on 30 March 2025).

The elucidation of the role of TXNIP in the pathogenesis of diabetes was decisive for its further investigation in its contribution to the development of GDM. Research on the link between TXNIP and GDM pathogenesis, although limited, is of growing interest. To date, observations have been performed on human placentas, cord blood, and maternal serum, as well as on cell models, like HTR-8/SVneo trophoblast cells mimicking GDM conditions. Given the restricted number of human studies in this field, in vitro experiments have provided valuable insights into the underlying molecular mechanisms of TXNIP dysregulation in GDM. These studies focus on molecular pathways implicated in conditions met in GDM pregnancies like oxidative stress, inflammation, and placental morphological alterations. TXNIP overexpression has been implicated in the pathophysiology of GDM, mainly through regulating inflammation and apoptosis, both of which are central mechanisms of GDM, finally contributing to placental dysfunction and impaired fetal development. Steps forward have also been made in the clarification of the mechanisms of TXNIP regulation involving *TXNIP* gene methylation and microRNAs, in GDM pregnancies, and particularly in placental tissue (Figure 3). On the other hand, little is known about the consequences of TXNIP overexpression in GDM offsprings [95].

### 5.1. TXNIP Expression in GDM

In normal human placental tissues obtained after legal abortion in different trimesters of pregnancy, TXNIP expression is relatively low during the first compared to the second and third trimesters, likely due to hypoxic conditions [96]. According to the published observations, TXNIP expression is unanimously higher in GDM compared to healthy pregnancies; however, it is not clear whether this overexpression is site specific. TXNIP has been found to be overexpressed in the syncytioctrophoblasts, cytotrophoblast cells, and trophoblasts [97,98,99], and downregulated in endothelial cells of placentas from GDM patients compared to healthy pregnancies [98]. Furthermore, a study on 20 pregnant insulin-treated GDM women (GDMA2) has shown that TXNIP and TRX expression were increased in maternal serum and placental tissue and decreased in umbilical cord blood compared to healthy individuals [100]. In addition, the TRX/TXNIP mRNA ratio was increased in placental tissue and umbilical cord blood compared to maternal serum [100]. Their findings support that the downregulation of TXNIP along with the upregulation of the TRX/TXNIP ratio in placental tissue and umbilical cord blood may represent a fetal protective mechanism for GDM-related oxidative stress. Consistent with these findings, Eren et al. [101] also suggested that TXNIP could mediate as part of a fetal compensatory mechanism to mitigate oxidative stress in GDM, revealing a higher TRX/TXNIP ratio in the serum of GDM patients during the early second trimester compared to controls. It has been suggested that the observed increase in TXNIP mRNA and the decreased protein expression levels in maternal serum from GDM patients indicate a possible post-transcriptional regulation of TXNIP expression [100,101].

### 5.2. Mechanisms of TXNIP Action in GDM

The mechanism by which glucose-induced TXNIP overexpression enacts its function has been investigated by in vitro analysis in the high glucose-induced TXNIP overexpression trophoblast cell line HTR-8/SVneo, which mimics GDM conditions. As described, it involves apoptotic and inflammatory signaling, similarly to beta cells. Thus, TXNIP upregulation promotes increased ROS accumulation, inhibits TRX expression, and promotes mitochondrial fragmentation, as well as cell apoptosis [97]. It should be noted that mitochondrial dysfunction is a key characteristic of GDM placentas, further contributing to placental dysfunction [97,102,103]. Another study on hyperglycemia-treated C17.2 neural stem cells has shown decreased expression levels of miR-17-5p, leading to upregulation of its target gene, TXNIP. The activation of the TXNIP/ASK1 pathway under hyperglycemic conditions promoted apoptosis in neural stem cells, contributing to neural cell dysfunction and increasing the risk of neural tube defects (NTDs) in offspring [104]. Further in vitro studies using miR-17-5p mimic-treated HTR-8/SVneo trophoblast cells revealed the inhibition of the TXNIP/NLRP3 pathway and enhanced trophoblast glucose consumption [105]. Importantly, cells transfected with the TXNIP vector resulted in upregulation of NLRP3 followed by impaired glucose consumption [105]. Hyperglycemia in GDM leads to reduced expression levels of miR-17-5p, upregulation of its target gene TXNIP, and subsequent activation of NLRP3 inflammasome, ultimately leading to the release of inflammatory cytokines and contributing to impaired trophoblast glucose uptake.

The effects of TXNIP in GDM-associated inflammatory pathways have been described by Pasternak et al. [100]. Their findings revealed significant upregulation of the pro-inflammatory TXNIP/TXN/STAT-3/SOCS3/NFƙB-p50 pathway in placental tissues from GDMA2 patients compared to healthy pregnancies [40]. Notably, NFƙB-p50 mRNA expression was significantly decreased in cord blood from GDMA2 patients compared to maternal tissues, further indicating a TXNIP-involved fetal protective response to the inflammatory environment of GDM [101]. In a recent study including 57 women with GDM, TXNIP levels in trophoblasts correlated positively with aortic intima-media thickness (aIMT), a well-known risk marker of cardiovascular disease in offspring [98]. However, TXNIP levels in umbilical/neonatal blood were not associated with GDM [98].

### 5.3. TXNIP and Trophoblast Dysfunction in GDM

Overexpression of TXNIP in HTR-8/SVneo trophoblast cells resulted in altered trophoblast morphology, and increased cell migration and invasion [99]. Further analysis into the underlying molecular mechanisms revealed that these effects were accompanied by abnormal expression of Epithelial–Mesenchymal Transition (EMT)-related factors, including Vimentin, as well as by upregulation of phosphorylated Signal Transducer and Activator of Transcription 3 (p-STAT3), an essential transcription factor for placental development [99]. Interestingly, these effects were reversed by the knockout of TXNIP [99]. These findings are consistent with previous studies revealing increased expression levels of p-STAT3 in placentas of GDMA2 mothers, further supporting the hypothesis that the STAT-3/TXNIP pathway is involved in GDM-related trophoblast dysfunction [100]. Conflicting results regarding trophoblast migration in GDM highlight the complexity of TXNIP’s role in GDM-related trophoblast dysfunction and emphasize the need for standardized methodologies to assess the role of TXNIP in GDM [97,99].

### 5.4. TXNIP and Placental Morphology in GDM

In placental tissues from obese and non-obese pregnant women, including a subgroup of 10 obese women with GDM, TXNIP expression levels have been found to be positively correlated with placental thickness and negatively correlated with placental surface area, both critical morphological factors related to the placenta’s efficiency to transfer oxygen and nutrients to the fetus [106], suggesting a relationship between placental oxidative status and placental alterations.

Autophagy is a key cellular prosess responsible for the regulation of placental development [107,108,109]. Dysregulation of autophagy has been observed in placental tissues from GDM patients compared to healthy pregnancies [110]. A study on HTR-8/SVneo trophoblast cells has shown that glucose-induced TXNIP upregulation resulted in reduced cell proliferation, increased apoptosis, and diminished autophagic activity, as indicated by dysregulation of autophagy markers [111]. On the other hand, knockdown of TXNIP in the same cell line effectively reversed these effects, restoring trophoblast viability and autophagy [111]. These findings suggest that TXNIP dysregulation plays a key role in GDM-associated trophoblast dysfunction, potentially through impairing autophagy. Table 1 summarizes the data obtained from research on TXNIP dysregulation in GDM-related pathophysiological mechanisms, including oxidative stress, inflammation, autophagy, apoptosis, and trophoblast behavior, further contributing to placental dysfunction.

### 5.5. TXNIP Methylation Status in GDM

Epigenome-Wide Association Studies (EWAS) have identified that GDM-related hyperglycemia could modify the offspring epigenome, ultimately contributing to adverse health effects [112]. Little is known about the role of TXNIP methylation status in GDM- related abnormalities. Houshmand-Oeregaard et al. [95] was the first study to investigate TXNIP methylation and expression levels in metabolic tissues of offspring from diabetic mothers. Their findings revealed increased methylation levels at CpG site cg19693031 of the *TXNIP* gene, accompanied by decreased *TXNIP* expression levels, in subcutaneous adipose tissue (SAT) of adult offspring from GDM mothers [95]. Interestingly, the unexpected *TXNIP* downregulation in SAT may reflect a protective compensatory mechanism of offspring to mitigate the risk of future GDM-related complications.

A subsequent meta-analysis revealed a negative correlation between TXNIP methylation levels in cord blood and maternal glucose levels, as mainly assessed by the area under the curve of glucose (AUC_gluc_) [113]. Interestingly, higher maternal AUC_gluc_ was associated with lower DNA methylation at two CpG sites within an exon of TXNIP (cg26974062 and cg02988288), though this correlation was observed exclusively in the non-GDM group [113]. It has been suggested that the absence of this association in GDM group could indicate the impact of GDM treatment, and particularly pharmacological intervention, on epigenetic regulation of TXNIP. Table 2 summarizes the associations between TXNIP expression or methylation and GDM-associated parameters, along with their implications.

## 6. Conclusions and Future Perspectives

The growing interest in investigating the pathophysiology of GDM is reflected in the approximately 3-fold increase in the number of observational and functional studies on GDM outcomes and placental signaling pathways over the last decade. Emerging evidence has identified TXNIP as a key player in GDM-related pathogenetic mechanisms, including oxidative stress, inflammation, apoptosis, impaired autophagy, and altered trophoblast behavior, these resulting in placental dysfunction and potential long term health effects for the fetus [97,99,104,105,111]. However, the complexity of the pathophysiology of GDM as well as the plethora of risk factors involved in the development of this complication make it difficult to unravel the role of TXNIP in GDM. The selection criteria of GDM women to be studied (age, diagnosis, BMI), the maternal glucose control during GDM treatment, the limited availability of maternal and fetal tissue samples from GDM pregnancies, and the lack of studies using GDM animal models are some of the limitations of secure outcome assessments. Undoubtedly, longitudinal studies will provide a better understanding of TXNIP’s role in GDM pathogenesis. In addition, further exploration into additional mechanistic studies is needed to evaluate the underlying molecular mechanisms of TXNIP dysregulation in GDM. Incorporating emerging technologies that enable precise quantification of post-translational modifications influencing TXNIP activity levels in GDM could provide valuable insights into the role of TXNIP in the pathophysiology of the disease.

Therapeutic modulators targeting TXNIP such as Verapamil are already being used to enhance beta cell function and improve diabetes-related complications [68]. In the same line, the study of the role of TXNIP in GDM pathophysiology aims to clarify the possibility of it serving as a therapeutic target. According to the data to date, the downregulation of TXNIP in GDM could potentially mitigate oxidative stress, reduce mitochondrial-related cell death, decrease inflammation, and improve trophoblast function. The discovery of the modulatory effect of miRNA-17-5p—using miR-17-5p mimic-treated HTR-8/SVneo trophoblast cells—on the TXNIP/NLRP3 pathway and trophoblast function and behavior reinforces this ambition. Undoubtety, further research is needed to assess the therapeutic potential, efficacy, and safety of TXNIP modulators in GDM-related complications.

## Figures and Tables

**Table 1:** Summary of published research regarding role of TXNIP expression in GDM-associated pathophysiological mechanisms.

Tissue/Cell Culture	TXNIP Expression Levels	TXNIP mRNA/Protein Expression Levels	Associated Parameters	Proposed Mechanism	Refs.
Placental tissue from GDM patients	↑	Protein	-	Oxidative stress, mitochondrial dysfunction, and apoptosis	[97]
HG-treated HTR-8/SVneo cells	↑	mRNA and protein	↑mitochondrial fragmentation, ↑caspase-3		
HTR-8/SVneo cells transfected with TXNIP vector (TXNIP overexpression)	↑	mRNA and protein	↓TRX, ↓cell proliferation, ↓cell migration, ↑caspase-3, ↑apoptotic cells, ↑ROS, ↑mitochondrial fragmentation, ↓mitochondrial membrane potential		
Placental tissue from GDM patients	↑	mRNA and protein	-	Impairment of trophoblast morphology through STAT-3 and Vimentin-related mechanisms	[99]
HTR-8/SVneo cells overexpressing TXNIP (Tet-on system)	↑	mRNA and protein	↑cell migration ability, ↑cell invasion ability, ↑densified cells, ↑Vimentin, ↑E-cadherin, ↓N-cadherin, ↑p-STAT3		
HTR-8/SVneo cells with TXNIP knockout (CRISPR-Cas9)	↓	mRNA and protein	↓cell migration ability, ↓cell invasion ability, ↑shrunken cells with disintegrated cytoplasm, ↓Vimentin, ↑N-cadherin, ↓E-cadherin, ↓p-STAT3		
Maternal blood from GDMA2 patients	↑	mRNA	↑TRX, ↑NFkΒ-50	Oxidative stress and inflammation	[100]
Placental tissue from GDMA2 patients	↑	Protein	↑TRX, ↑TRX/TXNIP ratio compared to maternal, ↑NFkΒ-50, ↑p-STAT3, ↑SOCS3	Fetal adaptations to GDM-related oxidative stress and inflammation	
Umbilical cord blood from GDMA2 patients	↓	mRNA	↓TRX compared to placenta, ↑TXN/TXNIP ratio- compared to maternal, ↓NFkΒ-50 compared to both maternal and placental		
Maternal serum from GDM patients	↓	Protein	↑TRX/TXNIP ratio	Fetal adaptations to GDM-related oxidative stress	[101]
E8.5 embryos from diabetic and non-diabetic mice	-	-	↓miR-17-5p	Apoptosis through ASK1 activation	[104]
HG-treated C17.2 neural stem cells	↑	mRNA and protein	↓miR-17-5p, ↓TRX/ASK1 complex, ↑ASK1 phosphorylation, ↑apoptotic cells, ↑cleaved caspase-3		
HG-treated C17.2 neural stem cells transfected with miR-17 mimic	↓	mRNA and protein	↑TRX/ASK1 complex, ↓ASK1 phosphorylation, ↓apoptotic cells, ↓cleaved caspase-3		
HG-treated C17.2 neural stem cells transfected with miR-17 inhibitor	↑	mRNA and protein	↓TRX/ASK1 complex, ↑ASK1 phosphorylation, ↑apoptotic cells, ↑cleaved caspase-3		
HG-treated C17.2 neural stem cells transfected with TXNIP siRNA (TXNIP Knockdown)	↓	mRNA and protein	↑TRX/ASK1 complex, ↓ASK1 phosphorylation, ↓apoptotic cells, ↓cleaved caspase-3		
HG-treated C17.2 neural stem cells transfected with TXNIP vector (TXNIP overexpression)	↑	mRNA and protein	↑TXNIP/TRX complex, ↓TXN/ASK1 complex, ↑ASK1 phosphorylation, ↑apoptotic cells, ↑cleaved caspase-3		
Placental tissue from GDM patients	↑	mRNA and protein	↑NLRP3, ↓miR-17-5p	Inflammation through NLRP3 inflammasome activation	[105]
HTR-8/SVneo cells transfected with TXNIP vector (TXNIP overexpression)	↑	mRNA and protein	↑NLRP3, ↓glucose consumption		
HTR-8/SVneo cells transfected with miR-17-5p mimic	↓	mRNA and protein	↓NLRP3, ↑glucose consumption		
HTR-8/SVneo cells co-transfected with miR-17-5p mimic and TXNIP vector	↑	mRNA and protein	↑NLRP3, ↓glucose consumption		
HG-treated HTR-8/SVneo cells	↑	mRNA and protein	↑NLRP3, ↓glucose consumption		
HG-treated HTR-8/SVneo cells transfected with miR-17-5p mimic	↓	mRNA and protein	↓NLRP3, ↑glucose consumption		
HG-treated HTR-8/SVneo cells	↑	mRNA	↑LDH, ↓cell viability, ↑apoptosis, ↓autophagosomes, ↑P62, ↓LC3-II/LC3-I ratio	Inhibition of autophagy	[111]
HG-treated HTR-8/SVneo cells transfected with siRNA TXNIP (TXNIP knockdown)	↓	mRNA	↓LDH, ↑cell viability, ↓apoptosis, ↑autophagosomes, ↓P62, ↑LC3-II/LC3-I ratio		
HTR-8/SVneo cells transfected with siRNA TXNIP (TXNIP knockdown)	↓	mRNA	↑autophagosomes, ↓P62, ↑LC3-II/LC3-I ratio		
HTR-8/SVneo cells transfected with siRNA TXNIP (TXNIP knockdown) and treated with autophagy inhibitor 3-MA	↓	mRNA	↓autophagosomes, ↑P62, ↓LC3-II/LC3-I ratio		
HG-treated HTR-8/SVneo cells transfected with siRNA TXNIP (TXNIP knockdown) and treated with 3-MA	↓	mRNA	↓cell viability, ↑apoptosis		

HG: Hyperglycemia; LDH: Lactate dehydrogenase; P62: Sequestosome 1; LC3-I/LC3-II: Microtubule-associated protein 1 light chain 3; si: Small-interference; 3-MA: 3-Methyladenine; GDM: Gestational diabetes mellitus; p-STAT3: phosphorylated-signal transducer and activator of transcription 3; TXNIP: Thioredoxin-Interacting Protein; TRX: Thioredoxin; ROS: Reactive Oxygen Species; GDMA2: Gestational Diabetes Mellitus Type 2; SOCS3: suppressor of cytokine signaling 3; NFkB: nuclear factor kappa B; NLRP3: nod-like receptor protein 3; ASK1: Apoptosis Signal-regulating Kinase 1; (↑): upregulation; (↓): downregulation.

**Table 2:** Reported associations between TXNIP expression or methylation and GDM-associated parameters, along with their implications.

Tissue/ Cell Culture	TXNIP Expression Levels	TXNIP mRNA/protein Expression Levels	Correlations	Implications	Refs.
Subcutaneous adipose tissue from offspring of GDM patients	↓	mRNA	TXNIP expression levels were negatively associated with TXNIP methylation levels	Offspring compensatory mechanisms against GDM-related complications	[95]
Placental tissue from GDM patients	↑trophoblasts and syncytiotrophoblasts ↓endothelial cells	Protein	TXNIP expression levels in trophoblasts were positively correlated with aIMT	Increased risk of cardiovascular disease in offsprings	[98]
Placental tissue from non-obese and obese patients, including a subgroup from obese patients with GDM	No difference	Protein	TXNIP expression levels were positively correlated with the placental thickness and negatively correlated with the placental surface	Decreased placental efficiency	[106]
Umbilical cord blood from pregnant women (metanalysis)	-	-	TXNIP methylation levels were negatively correlated with AUC_gluc_ exclusively in non-GMD groups	Impact of maternal glycemic control on the epigenetic regulation of TXNIP	[113]

GDM: gestational diabetes mellitus; TXNIP: Thioredoxin-Interacting Protein; AUC_gluc_: area under the curve of glucose; aIMT: aorta intima-media thickness; (↑): upregulation; (↓): downregulation.

## Data Availability

No new data were created or analyzed in this study.
